# Cognitive and emotional pathways to diabetes distress: a structural equation and latent profile analysis

**DOI:** 10.3389/fmed.2026.1870366

**Published:** 2026-06-19

**Authors:** Bandar S. Alharbi, Majed M. Aljabri, Endale Alemayehu Ali

**Affiliations:** 1 Community and Psychiatric Mental Health Nursing Department, College of Nursing, King Saud University, Riyadh, Saudi Arabia; 2 Department of Public Health and Primary Care, KU Leuven, Leuven, Belgium

**Keywords:** diabetes distress, illness perception, latent profile analysis, pain catastrophizing, psychological burden, structural equation modeling

## Abstract

**Background:**

Diabetes distress is a common and clinically significant condition linked to the daily demands of diabetes management. It affects up to 36% of individuals with type 2 diabetes and is associated with poor outcomes. However, the cognitive and emotional mechanisms underlying this distress remain unclear, especially in high-burden settings such as Saudi Arabia. The study aims to quantify the cognitive and emotional pathways associated with diabetes distress and identify distinct profiles of psychological burden.

**Methods:**

A cross-sectional study was conducted among 438 adults with diabetes in 4 primary healthcare centers in Saudi Arabia. Participants were recruited using convenience sampling, and data were collected through a structured questionnaire administered during healthcare visits. Pain catastrophizing, illness perceptions, and diabetes distress were assessed using validated scales. We used structural equation modeling (SEM) to estimate direct and indirect effects and latent profile analysis (LPA) to identify patient subgroups. Multinomial logistic regression was used to examine associated factors.

**Results:**

The findings showed that pain catastrophizing (*β* = 0.30; 95% CI: 0.19–0.44) and emotional representations of illness (*β* = 0.51; 95% CI: 0.17–0.83) were positively associated with diabetes distress. Conversely, perceived consequences did not show a significant association. Pain catastrophizing was strongly associated with both perceived consequences (*β* = 0.68; 95% CI: 0.57–0.76) and emotional representations (*β* = 0.66; 95% CI: 0.60–0.81). The indirect effect of pain catastrophizing on diabetes distress through emotional representations was significant (*β* = 0.34; 95% CI: 0.11–0.59), while through perceived consequences was not (*β* = 0.06; 95% CI: −0.17 to 0.30). The total effect of pain catastrophizing on distress was strong (*β* = 0.70; 95% CI: 0.63–0.83). The SEM explained 67% of the variance in diabetes distress. The study identified three profiles: low burden, moderate distress, and high burden. A higher burden was associated with a longer disease duration and complications, while higher education reduced risk.

**Conclusion:**

Diabetes distress is driven by strong cognitive and emotional pathways, with emotional illness representations acting as the primary mediator. Distinct patient profiles highlight the need for targeted interventions.

## 1 Introduction

Diabetes mellitus is a chronic disease that poses a major global public health challenge (1, 2). It affects more than 589 million adults worldwide, with numbers expected to rise steadily in the coming decades (3). The negative emotional experiences that arise from the ongoing psychosocial demands of living with diabetes lead to diabetes distress (4). Diabetes distress refers to the emotional burdens, worries, and concerns specifically associated with living with and managing diabetes, including the demands of self-care, fear of complications, interactions with healthcare providers, and the impact of diabetes on interpersonal relationships (5, 6). It emerges as a persistent concern due to the fear of future complications and feelings of being overwhelmed by the need for continuous vigilance (7). As patients frequently report frustration with self-management demands and feelings of being overwhelmed, these experiences accumulate over time and can intensify as the disease progresses. Existing evidence shows that higher diabetes distress is associated with poor glycemic control, reduced adherence to treatment, and an increased risk of complications (8, 9).

Diabetes distress has substantial clinical implications as it is directly associated with a greater symptom burden, reduced functional capacity, and increased work disability. It also increases overall healthcare costs as patients with higher levels of distress tend to use more healthcare services. Attention to diabetes distress has increased in recent years due to its high prevalence and impact on disease management. A meta-analysis reported that approximately 36% of individuals with type 2 diabetes experience significant levels of distress (10). This high prevalence, combined with its strong association with poor adherence and glycemic control, underscores the need to better understand the underlying mechanisms and to identify patients at the highest risk.

Cognitive and emotional processes are the main factors in shaping this distress (11). Pain catastrophizing, defined as an exaggerated negative cognitive and emotional response to actual or anticipated pain, is characterized by rumination, magnification, and feelings of helplessness (12, 13). The emotional representation domain of illness perception reflects the emotional reactions individuals have toward their illness, including worry, fear, and emotional distress. In this study, emotional representations were assessed using three items evaluating worry about diabetes, fear of complications, and the extent to which diabetes causes emotional distress. Although diabetes is not primarily a pain condition, many individuals experience pain related to diabetes complications, comorbid chronic conditions, or treatment-related burdens. Moreover, pain catastrophizing reflects a broader maladaptive cognitive style that may extend beyond pain experiences and influence perceptions of illness and emotional responses to diabetes. Studies have shown that in the majority of cases, healthcare providers and patients are not clearly aware of this emotional distress, and it becomes a serious concern due to its link to poor glycemic control (14, 15).

The diabetes burden has increased rapidly in Asian countries, driven by urbanization, lifestyle changes, and population aging. A recent meta-analysis of diabetes distress in South Asian countries reported a pooled prevalence of 44%, with the highest prevalence observed in Pakistan (16). They also identified emotional burden as the most prevalent domain (60%). However, few studies have applied advanced modeling approaches to disentangle the direct and indirect pathways linking cognitive and emotional factors to distress. The prevalence of diabetes in the Middle East is also among the highest globally. Sociocultural factors, including family structure, health beliefs, and stigma, influence how individuals perceive and cope with chronic illness (17). However, research remains fragmented. Many studies focus on prevalence or simple associations without examining underlying mechanisms or heterogeneity across patient groups.

Studies reveal that Saudi Arabia has a high prevalence of diabetes, ranking among the top 10 countries globally for prevalence among adults aged 20–79 years (18). The healthcare system has made significant investments in diabetes management, but psychological aspects of care remain under-addressed. There is limited evidence on how cognitive processes, such as catastrophizing, interact with illness perceptions to shape distress. In addition, population heterogeneity is rarely explored. Patients are often treated as a homogeneous group, despite clear differences in clinical status, education, and disease duration.

Therefore, our study addresses several important gaps identified in previous studies. Structural equation modeling was used to quantify cognitive and emotional pathways linking pain catastrophizing, illness perceptions, and diabetes distress. This allowed us to test both direct effects and mediation mechanisms within a unified framework. In addition, latent profile analysis was used to identify distinct subgroups of patients based on their psychological burden. This approach captures the heterogeneity that is often missed in conventional analyses. Finally, this study examined sociodemographic and clinical factors associated with profile membership to identify high-risk groups.

## 2 Methods

### 2.1 Study design and sampling

This cross-sectional study was conducted to examine the cognitive and emotional pathways associated with diabetes distress and to identify latent patient profiles based on these constructs. The study population comprised adults with diabetes attending four primary healthcare centers in Riyadh, Saudi Arabia. This study aimed to capture psychological and clinical characteristics relevant to diabetes management within routine primary care settings.

Data were collected between 1 February and 28 February 2026. An Arabic-language survey was administered electronically using Google Forms. Eligible participants accessed the questionnaire by scanning a QR code provided on the informed consent form. The survey was promoted through notice boards in the participating primary healthcare centers and further disseminated by nursing staff during routine patient visits. Participants completed the questionnaire independently; however, nursing staff were available to clarify study procedures and questionnaire instructions upon request. No assistance was provided in selecting or interpreting responses. Information regarding the use of such assistance was not systematically recorded.

Participants were adults aged 18 years or older with a confirmed diagnosis of diabetes mellitus who were able to provide informed consent and complete the study questionnaire. Patients with severe cognitive impairment or conditions that could limit their ability to provide reliable responses were excluded. A total of 502 individuals responded to the survey. After excluding records with missing data and responses that did not meet the eligibility criteria, 438 participants were included in the final analysis, corresponding to a response rate of 87.3%.

### 2.2 Data collection and measures

A structured questionnaire was used to collect data, which included validated instruments assessing pain catastrophizing, illness perceptions, and diabetes distress, as well as sociodemographic and clinical characteristics.

Pain catastrophizing was evaluated using the validated Pain Catastrophizing Scale (PCS), comprising 13 questions about rumination, magnification, and helplessness in relation to pain experience (19, 20). Each item is rated on a 5-point Likert scale ranging from 0 (“not at all”) to 4 (“all the time”). For this study, an overall PCS score was calculated by averaging responses across all 13 items, resulting in a score ranging from 0 to 4. Higher scores indicate greater levels of pain catastrophizing.

Perceptions about illness were evaluated using specific domains of the Illness Perception Questionnaire (IPQ) (21, 22). The consequences domain included three questions evaluating the impact and severity of diabetes on everyday functioning. Emotional representation consisted of three questions assessing feelings of fear and worry associated with the disease. Diabetes distress was assessed using the Diabetes Distress Scale (DDS), which includes 17 items capturing emotional burden, regimen-related distress, and interpersonal distress (5, 6). A total distress score was calculated as the mean of all items, with higher scores indicating greater distress. In assessing these validated instruments, minor wording adjustments were applied to enhance their cultural relevance and contextual clarity for participants in Saudi Arabia. These adaptations were carefully implemented to preserve the original meaning and psychometric properties of the scales. Sociodemographic variables were also accounted for, including age, education level, and clinical variables, including years since diabetes diagnosis and the presence of diabetes-related complications.

### 2.3 Reliability assessment

Internal consistency of each scale was evaluated using Cronbach’s alpha. The PCS and DDS demonstrated excellent reliability (*α* = 0.96), while the IPQ domains showed good reliability (α = 0.86). These results supported the use of composite scores in subsequent analyses.

### 2.4 Data analysis

#### 2.4.1 Structural equation modeling

We used structural equation modeling (SEM) to examine the hypothesized cognitive–emotional pathways linking pain catastrophizing, illness perceptions, and diabetes distress. A measurement model was first specified, with PCS, IPQ consequences, IPQ emotional representations, and DDS modeled as latent variables, each indicated by its respective items.

The structural model specified direct paths from pain catastrophizing to diabetes distress and to both illness perception domains. Additional paths were specified from illness perceptions to diabetes distress. Covariance between the two illness perception domains was included to account for shared variance. Indirect effects were defined to assess mediation pathways through illness perceptions.

The model was estimated using maximum likelihood estimation with robust standard errors (MLR) to account for potential non-normality. Missing data were handled using full information maximum likelihood (FIML). Model fit was evaluated using multiple indices, including the comparative fit index (CFI), Tucker–Lewis index (TLI), root mean square error of approximation (RMSEA), and standardized root mean square residual (SRMR). Results were presented as standardized path coefficients (*β*) with their corresponding 95% confidence intervals.

#### 2.4.2 Latent profile analysis

Latent profile analysis (LPA) was conducted to identify groups of participants with similar patterns of pain catastrophizing, illness perceptions, and diabetes distress. This person-centered approach allows individuals with comparable psychological characteristics to be grouped into distinct profiles. Several models containing between one and four profiles were evaluated. The final model was selected based on statistical indicators of model fit and the interpretability of the identified profiles.

A three-profile solution provided the most meaningful classification of participants. Profile labels were assigned according to the observed levels of pain catastrophizing, illness perceptions, and diabetes distress within each group. The low-burden profile was characterized by consistently low scores across all psychological measures, the moderate-distress profile showed intermediate scores, and the high-burden profile exhibited the highest levels of pain catastrophizing, negative illness perceptions, and diabetes distress. These labels were descriptive and were used solely to facilitate the interpretation of the identified participant groups.

#### 2.4.3 Multinomial regression analysis

After identifying the latent profiles, multinomial logistic regression was used to examine which participant characteristics were associated with assignment to these profiles. In other words, the analysis evaluated factors associated with belonging to the moderate-distress or high-burden profiles compared with the low-burden profile. The low-burden profile served as the reference group. Age, educational level, years since diabetes diagnosis, and the presence of diabetes-related complications were included as explanatory variables based on their potential influence on psychological wellbeing and diabetes management.

The analysis estimated the likelihood of belonging to the moderate-distress or high-burden profiles relative to the low-burden profile. Results are reported as relative risk ratios (RRRs) with 95% confidence intervals. An RRR greater than 1 indicated a higher likelihood of belonging to a given profile, whereas an RRR less than 1 indicated a lower likelihood.

### 2.5 Software

All analyses were conducted using R statistical software (version 4.2.1) (23). Packages used included *lavaan* for structural equation modeling, *tidyLPA* for latent profile analysis, and *nnet* for multinomial regression.

## 3 Results

A total of 502 individuals responded to the survey. After excluding incomplete responses and records with missing information required for the analysis, 438 participants were included in the final study sample, corresponding to a response rate of 87.3%.

The descriptive results showed a consistent gradient across the three profiles (low burden, moderate distress, and high burden), with clear differences in demographic and clinical characteristics (Table 1). Participants in the higher burden group were mostly older. The proportion aged 45–54 was highest in this group (31.0%), and older age categories, particularly ≥65 years, were more represented than in the low-burden group. In contrast, the low-burden group was more concentrated in the middle-aged range (35–54 years), with fewer older individuals. A clear pattern was also observed in education, with the low-burden group having the highest proportion of participants with a bachelor’s degree (50.9%). On the other hand, the high-burden group had fewer individuals at this level (29.8%) and a higher proportion with lower educational attainment, including less than high school education (20.2%). Similarly, the difference persisted across years since diagnosis. Approximately half of the low-burden group had been diagnosed for less than 1 year (46.4%), while this proportion dropped sharply in the high-burden group (17.9%). In contrast, long disease duration (>10 years) was most common in the high-burden group (32.1%) and least common in the low-burden group (15.2%). A clear difference is also observed in diabetes complications, with 8.9% of the low-burden group reporting complications, compared to 21.1% in the moderate group and 41.7% in the high-burden group. Conversely, the absence of complications was highest in the low-burden group (91.1%) and lowest in the high-burden group (58.3%).

**Table 1:** Distribution of sociodemographic and clinical characteristics across latent psychological burden profiles among adults with diabetes.

Variable	Category	Low burden (*n* = 112)	Moderate distress (*n* = 242)	High burden (*n* = 84)
Age (years)	18–24	2 (1.8%)	10 (4.1%)	4 (4.8%)
	25–34	23 (20.5%)	46 (19.0%)	11 (13.1%)
	35–44	32 (28.6%)	42 (17.4%)	17 (20.2%)
	45–54	32 (28.6%)	60 (24.8%)	26 (31.0%)
	55–64	18 (16.1%)	49 (20.2%)	17 (20.2%)
	≥65	5 (4.5%)	35 (14.5%)	9 (10.7%)
Education level	Less than high school	5 (4.5%)	41 (16.9%)	17 (20.2%)
	High school	33 (29.5%)	83 (34.3%)	30 (35.7%)
	Diploma	14 (12.5%)	19 (7.9%)	10 (11.9%)
	Bachelor	57 (50.9%)	87 (36.0%)	25 (29.8%)
	Graduate	3 (2.7%)	12 (5.0%)	2 (2.4%)
Years since diagnosis	<1 year	52 (46.4%)	52 (21.5%)	15 (17.9%)
	1–5 years	25 (22.3%)	84 (34.7%)	26 (31.0%)
	6–10 years	18 (16.1%)	45 (18.6%)	16 (19.0%)
	>10 years	17 (15.2%)	61 (25.2%)	27 (32.1%)
Diabetes complications	No	102 (91.1%)	191 (78.9%)	49 (58.3%)
	Yes	10 (8.9%)	51 (21.1%)	35 (41.7%)

We have assessed the internal consistency of scales used in this study and found good internal consistency (Supplementary Table S1). The PCS (*α* = 0.96) and Diabetes Distress Scale (α = 0.96) showed excellent reliability, indicating very high internal coherence among items. The illness perception subscales also showed good reliability, with α = 0.86 for perceived consequences and α = 0.84 for emotional representations.

We performed a model fit check of the structural equation model and found a good model fit to the data (*χ*^2^(246) = 555.5, *p* < 0.001; CFI = 0.940; TLI = 0.932; RMSEA = 0.067; SRMR = 0.038), indicating that the proposed cognitive–emotional framework adequately represents the observed relationships.

The results for the direct and indirect associations of cognitive and emotional pathways and diabetes distress are presented in Table 2. The findings show that pain catastrophizing was significantly and positively associated with diabetes distress (DDS) (*β* = 0.30, 95% CI: 0.19–0.44, *p* < 0.001). In addition, emotional illness perceptions were independently associated with distress (*β* = 0.51, 95% CI: 0.17–0.83, *p* = 0.003). In contrast, perceived consequences of diabetes were not significantly associated with distress (*β* = 0.09, 95% CI: −0.26 to 0.44, *p* = 0.598). A strong association was found between pain catastrophizing and both domains of illness perception. Pain catastrophizing was strongly associated with perceived consequences (*β* = 0.68, 95% CI: 0.57–0.76, *p* < 0.001) and emotional representations (*β* = 0.66, 95% CI: 0.60–0.81, *p* < 0.001). This indicates that individuals with higher catastrophizing tend to perceive their illness as more severe and emotionally distressing.

**Table 2:** Structural equation model of cognitive and emotional pathways to diabetes distress.

Pathway	*β* (95% CI)	*p*-value
Direct effects		
PCS → DDS	0.30 (0.19, 0.44)	<0.001
IPQ emotional → DDS	0.51 (0.17, 0.83)	0.003
IPQ consequences → DDS	0.09 (−0.26, 0.44)	0.598
PCS → IPQ consequences	0.68 (0.57, 0.76)	<0.001
PCS → IPQ emotional	0.66 (0.60, 0.81)	<0.001
Indirect effects		
PCS → IPQ emotional → DDS	0.34 (0.11, 0.59)	0.004
PCS → IPQ consequences → DDS	0.06 (−0.17, 0.30)	0.600
Total effect		
PCS → DDS (total)	0.70 (0.63, 0.83)	<0.001

PCS, Pain Catastrophizing Scale, IPQ, Illness Perception Questionnaire, DDS, Diabetes Distress Scale, β, standardized coefficient, CI, confidence interval.

Mediation analysis revealed that the association between pain catastrophizing and diabetes distress operates primarily through emotional representations. The indirect pathway via emotional representations was statistically significant (*β* = 0.34, 95% CI: 0.11–0.59, *p* = 0.004), whereas the pathway through perceived consequences was not (*β* = 0.06, 95% CI: −0.17–0.30, *p* = 0.60). This indicates that the emotional interpretation of the illness, rather than the cognitive evaluation of its consequences, is the mechanism linking catastrophizing to distress. There was a strong and positive total effect of pain catastrophizing on diabetes distress (*β* = 0.70, 95% CI: 0.63–0.83, *p* < 0.001), reflecting the combined direct and indirect pathways.

The structural equation model explained 67% of the variance in diabetes distress, with pain catastrophizing accounting for 46% of the variance, and perceived consequences and emotional representations accounting for 44% each (Supplementary Table S2). The findings suggest that pain catastrophizing and illness perceptions together capture most of the variability in distress, strongly supporting the adequacy of the proposed cognitive–emotional framework.

The profile characteristics show a clear and consistent gradient of psychological burden across groups (Table 2). Individuals in the low-burden profile had uniformly low scores across all domains: pain catastrophizing (PCS = 0.58), illness perceptions (IPQ consequences = 1.42; IPQ emotional = 1.38), and diabetes distress (DDS = 1.41). This group also had the lowest prevalence of diabetes complications (8.9%), indicating a relatively stable clinical and psychological status. The moderate distress group showed intermediate levels across all measures. PCS increased to 1.33, illness perceptions were elevated (IPQ consequences = 2.80; IPQ emotional = 2.89), and DDS increased to 2.50. The prevalence of complications also increased to 21.1%. The high-burden profile exhibited substantially increased scores across all domains. PCS reached 2.55, and both cognitive and emotional illness perceptions were significantly higher (IPQ consequences = 4.04; IPQ emotional = 4.10). Diabetes distress was also highest (DDS = 3.58). Importantly, this group had the highest prevalence of complications (41.7%), approximately five times that of the low-burden group. Overall, we observed a strong monotonic increase in pain catastrophizing, negative illness perceptions, diabetes distress, and complication prevalence across all profiles. This pattern supports the validity of the latent profiles and indicates that psychological burden in diabetes is closely aligned with both cognitive-emotional processes and clinical severity.

Model fit improved with an increasing number of profiles, as shown by decreasing AIC, BIC, and SABIC values from the 1-profile to the 4-profile solution (Supplementary Table S3). The largest improvement occurred with the 3-profile model, indicating substantial gains in fit. Although the 4-profile model yielded the lowest information criteria, the improvement over the 3-profile model was modest relative to the increase in model complexity. Therefore, to balance statistical fit and interpretability, we selected the 3-profile solution as the optimal model (see Table 3).

**Table 3:** Characteristics of latent psychological burden profiles based on pain catastrophizing, illness perceptions, and diabetes distress.

Characteristic	Low burden (*n* = 112)	Moderate distress (*n* = 242)	High burden (*n* = 84)
PCS score, mean	0.58	1.33	2.55
IPQ consequences, mean	1.42	2.80	4.04
IPQ emotional, mean	1.38	2.89	4.10
DDS score, mean	1.41	2.50	3.58
Diabetes complications, %	8.9	21.1	41.7

A clear and consistent pattern was observed in the factors associated with psychological burden profiles (Table 4). We found that compared with individuals in the low-burden group, age was not a strong predictor of profile membership. Most age categories showed reduced relative risk ratios for both moderate distress and high burden, with wide confidence intervals. Only those aged 35–44 years had a significantly lower likelihood of belonging to the moderate-distress group. This suggests that younger adults may experience relatively higher distress compared with this group, although this pattern was not consistent across other age categories. A strong association was found for educational attainment, particularly for the moderate distress profile. Individuals with diploma-level education (RRR = 0.22, 95% CI: 0.06–0.76) and bachelor’s degrees (RRR = 0.27, 95% CI: 0.09–0.79) were significantly less likely to belong to the moderate-distress group than those with lower education. A similar pattern was observed for the high-burden group, where individuals with a bachelor’s degree had a substantially reduced likelihood (RRR = 0.20, 95% CI: 0.06–0.69). These findings indicate that higher education may buffer against psychological burden in diabetes.

**Table 4:** Multinomial logistic regression of factors associated with latent psychological burden profiles (reference: low burden).

Characteristic	Moderate distress vs low RRR (95% CI)	High burden vs low RRR (95% CI)
Intercept	9.60 (1.45, 63.43)	3.56 (0.42, 30.45)
Age		
25–34	0.29 (0.06, 1.54)	0.17 (0.02, 1.17)
35–44	0.17 (0.03, 0.92)	0.16 (0.02, 1.11)
45–54	0.23 (0.04, 1.22)	0.17 (0.03, 1.19)
55–64	0.28 (0.05, 1.56)	0.16 (0.02, 1.18)
≥65	0.46 (0.07, 3.23)	0.16 (0.02, 1.51)
Education level		
High school	0.47 (0.16, 1.40)	0.48 (0.14, 1.64)
Diploma	0.22 (0.06, 0.76)	0.28 (0.07, 1.18)
Bachelor	0.27 (0.09, 0.79)	0.20 (0.06, 0.69)
Graduate	0.79 (0.15, 4.09)	0.27 (0.03, 2.37)
Years since diagnosis		
1–5 years	3.98 (2.14, 7.39)	4.07 (1.76, 9.42)
6–10 years	2.45 (1.17, 5.10)	2.81 (1.05, 7.54)
>10 years	2.28 (1.05, 4.95)	3.54 (1.31, 9.53)
Diabetes complications (Yes)	2.32 (1.08, 4.97)	6.26 (2.73, 14.33)

Relative Risk Ratios (RRR) and 95% confidence intervals (CI) are reported. The reference group for the outcome is the low burden profile. Reference categories for predictors are: age 18–24 years, less than high school education, <1 year since diagnosis, and no diabetes complications.

The findings show a strong association with years since diagnosis. Compared with individuals diagnosed within the past year, those with longer disease duration had a significantly higher likelihood of belonging to both moderate- and high-burden profiles. For example, individuals with 1–5 years since diagnosis had approximately a 4-fold higher risk of moderate distress (RRR = 3.98, 95% CI: 2.14–7.39) and high burden (RRR = 4.07, 95% CI: 1.76–9.42). This elevated risk persisted for longer durations, including more than 10 years since diagnosis, highlighting the cumulative psychological impact of living with diabetes over time.

We also found a strong association with diabetes-related complications. Individuals with complications were more than twice as likely to belong to the moderate-distress group (RRR = 2.32, 95% CI: 1.08–4.97) and to the high-burden group (RRR = 6.26, 95% CI: 2.73–14.33). This indicates that complications are a major driver of increased psychological burden.

## 4 Discussion

This study examined the cognitive and emotional pathways driving diabetes distress and identified distinct psychological burden profiles among adults with diabetes in Saudi Arabia. The findings showed that pain catastrophizing had both direct and indirect associations with diabetes distress. The total effect of pain catastrophizing on diabetes distress was substantial (*β* = 0.70), indicating that higher levels of catastrophizing were linked to greater distress. The study also found that emotional—rather than cognitive evaluations of disease consequences—played a central mediating role. We identified three clear profiles of psychological burden, with strong gradients across clinical and sociodemographic characteristics.

The strong direct association between pain catastrophizing and diabetes distress aligns with established evidence in chronic disease research. Pain catastrophizing reflects a maladaptive cognitive style characterized by rumination, magnification, and helplessness. A recent longitudinal study showed that increased catastrophizing was associated with elevated pain interference, resulting in increased depressive symptoms over time (24). In the context of diabetes, this cognitive pattern likely amplifies the perceived challenges of self-management, leading to increased emotional strain (25). Our findings extend this evidence by quantifying both direct and mediated pathways within a single model.

We found that emotional representations had a strong independent association with diabetes distress and mediated a substantial proportion of the effect of catastrophizing. In contrast, the perceived consequences of diabetes were not significantly associated with distress. This distinction suggests that how patients feel about their illness drives distress more than how they evaluate its severity. Our finding is consistent with the Common-Sense Model of self-regulation (26). Previous studies have further demonstrated that emotional responses such as fear, anxiety, and worry are stronger predictors of distress and poor outcomes than cognitive beliefs alone (27, 28). Our results confirm and extend these findings in the context of diabetes by demonstrating a clear mediation pathway. In our study, the high explained variance in diabetes distress, reaching 67%, indicates that the model captures important determinants of psychological burden. The use of structural equation modeling allowed the simultaneous estimation of complex pathways and reduced measurement error. The strong association between high-burden profiles and complications aligns with previous evidence. Patients with higher distress are more likely to experience complications and poor outcomes (29). The association with longer disease duration reflects cumulative psychological burden over time (30).

Previous studies have demonstrated that diabetes distress can be reduced through a range of interventions, including diabetes self-management education, cognitive behavioral therapy, problem-solving therapy, mindfulness-based interventions, and collaborative care approaches (31–33). These interventions aim to improve coping skills, emotional regulation, self-efficacy, and diabetes self-management behaviors. The present findings extend this literature by suggesting that interventions targeting maladaptive cognitive processes, particularly pain catastrophizing and negative emotional illness perceptions, may be beneficial.

### 4.1 Mechanistic implications

Our findings support a clear mechanism. Pain catastrophizing shapes how patients interpret their illness, increasing negative emotional representations, such as fear and worry (34). These emotional responses subsequently drive diabetes distress. From a practical standpoint, interventions that target catastrophizing, such as cognitive behavioral therapy, may reduce distress by modifying maladaptive thought patterns (35).

Addressing emotional illness perceptions through educational and psychological support interventions, alongside emotional regulation strategies, may have a greater impact on reducing diabetes distress than focusing solely on disease knowledge.

The lack of association with perceived consequences suggests that increasing awareness of disease severity alone is insufficient. Educational interventions that focus only on complications may fail to reduce distress and could even increase it if they amplify fear without providing coping strategies.

### 4.2 Implications for practice and future study

These findings support targeted, mechanism-based interventions. Clinicians should assess both cognitive distortions and emotional responses in patients with diabetes. Screening for diabetes distress should be integrated into routine care. Patients in the high-burden profile require more intensive psychological support.

Future studies should use longitudinal designs to confirm the causal pathways. Intervention studies should test whether modifying catastrophizing and emotional perceptions reduces distress and improves clinical outcomes. Expanding this approach to other regions will strengthen its generalizability. Our study provides clear evidence that diabetes distress is driven by interconnected cognitive and emotional processes. Addressing these mechanisms offers a direct path to improving patient outcomes.

### 4.3 Strengths and limitations

Our study has several strengths. It applies a coherent analytical approach that integrates structural equation modeling with latent profile analysis. This approach captures both underlying mechanisms and population heterogeneity within a single study. The study relies on validated and widely used instruments, including the PCS, the Illness Perception Questionnaire, and the Diabetes Distress Scale. All measures demonstrated high internal consistency, which supports the reliability of the constructs. We collected the data in real-world primary healthcare settings in Saudi Arabia, which strengthens the practical relevance of the findings. The combination of cognitive, emotional, and clinical variables provides a comprehensive assessment of diabetes-related psychological burden. The findings also contribute context-specific evidence from a region with high diabetes prevalence and limited prior studies on psychological mechanisms.

We acknowledge several limitations. With the cross-sectional design of this study, we cannot conclude the causal effect. The directionality of associations cannot be fully established, and reverse or bidirectional relationships are possible. As all data were self-reported, there is a possibility of reporting bias, including social desirability and recall bias. Psychological constructs such as distress and catastrophizing may be influenced by current mood or context at the time of survey completion. The use of self-administered questionnaires may also lead to misinterpretation of items, particularly among participants with lower educational levels. Selection bias is possible due to the recruitment strategy. Participants were recruited from primary healthcare centers and completed the survey voluntarily through a QR-based system. This may exclude individuals with limited digital literacy, lower engagement with healthcare services, or more severe conditions that limit participation. There may also be residual confounding effects. Although we accounted for several sociodemographic and clinical variables, other relevant factors were not measured. Measurement overlap between constructs may also be present. In addition, the study population included individuals with varying demographic and clinical characteristics, contributing to heterogeneity within the sample. Consequently, the findings may not be fully representative of the broader population of adults with diabetes. Emotional illness perceptions and diabetes distress both capture affective experiences, which may inflate associations. Although the modeling approach accounts for latent structures, conceptual overlap cannot be fully excluded. In addition, cultural adaptation of psychological scales may influence how participants interpret and respond to items.

## Data Availability

The raw data supporting the conclusions of this article will be made available by the authors, without undue reservation.
