# Supplementary material for: Cognitive and emotional pathways to diabetes distress: a structural equation and latent profile analysis
Source: Front Med (Lausanne). 2026 Jun 19;13:1870366. doi: 10.3389/fmed.2026.1870366 (PMC13327860; doi:10.3389/fmed.2026.1870366)
Supplement: Supplementary file 1 [file Table_1.DOCX]

**Suppmementary Materials**

**Cognitive and Emotional Pathways to Diabetes Distress: A Structural Equation and Latent Profile Analysis**

Bandar S. Alharbi^1*^, Majed M. Aljabri^1^, Endale Alemayehu Ali^2^*

^1^Community and Psychiatric Mental Health Department, College of Nursing, King Saud University, Riyadh 12375, Saudi Arabia

^2^Department of Public Health and Primary Care, KU Leuven, Kapucijnenvoer 33, 3000 Leuven, Belgium

^*^ Shared corresponding author (Email: [banalharbi@ksu.edu.sa](mailto:banalharbi@ksu.edu.sa); [endalestat@gmail.com](mailto:endalestat@gmail.com))

Table S1. Internal consistency reliability of study scales used to assess pain catastrophizing, illness perceptions, and diabetes distress

| Scale | Items (n) | Cronbach’s α | 95% CI | Mean (SD) |
| --- | --- | --- | --- | --- |
| Pain Catastrophizing Scale (PCS) | 13 | 0.96 | 0.95–0.96 | 1.40 (0.96) |
| Illness Perception (Consequences) | 3 | 0.86 | 0.84–0.88 | 2.70 (1.00) |
| Illness Perception (Emotional) | 3 | 0.84 | 0.81–0.87 | 2.70 (1.10) |
| Diabetes Distress Scale (DDS) | 17 | 0.96 | 0.95–0.96 | 2.40 (1.00) |

Table S2. Variance explained by the structural model

| Latent variable | R² |
| --- | --- |
| Diabetes Distress (DDS) | 0.67 |
| IPQ – Consequences | 0.46 |
| IPQ – Emotional | 0.44 |

Table S3. Model fit indices for latent profile analysis solutions (1–4 profiles)

| Number of profiles | Log-likelihood | Parameters | AIC | BIC | SABIC |
| --- | --- | --- | --- | --- | --- |
| 1 | −2529 | 8 | 5073 | 5106 | 5080 |
| 2 | −2233 | 13 | 4491 | 4544 | 4503 |
| 3 | −2080 | 18 | 4196 | 4269 | 4212 |
| 4 | −2043 | 23 | 4132 | 4226 | 4153 |
